# Functional Changes in Littoral Macroinvertebrate Communities in Response to Watershed-Level Anthropogenic Stress

**DOI:** 10.1371/journal.pone.0101499

**Published:** 2014-07-09

**Authors:** Katya E. Kovalenko, Valerie J. Brady, Jan J. H. Ciborowski, Sergey Ilyushkin, Lucinda B. Johnson

**Affiliations:** 1 University of Windsor, Windsor, Ontario, Canada; 2 Natural Resources Research Institute, University of Minnesota Duluth, Duluth, Minnesota, United States of America; 3 Colorado School of Mines, Golden, Colorado, United States of America; Texas Tech University, United States of America

## Abstract

Watershed-scale anthropogenic stressors have profound effects on aquatic communities. Although several functional traits of stream macroinvertebrates change predictably in response to land development and urbanization, little is known about macroinvertebrate functional responses in lakes. We assessed functional community structure, functional diversity (Rao’s quadratic entropy) and voltinism in macroinvertebrate communities sampled across the full gradient of anthropogenic stress in Laurentian Great Lakes coastal wetlands. Functional diversity and voltinism significantly decreased with increasing development, whereas agriculture had smaller or non-significant effects. Functional community structure was affected by watershed-scale development, as demonstrated by an ordination analysis followed by regression. Because functional community structure affects energy flow and ecosystem function, and functional diversity is known to have important implications for ecosystem resilience to further environmental change, these results highlight the necessity of finding ways to remediate or at least ameliorate these effects.

## Introduction

Widespread anthropogenic modification of the landscape is jeopardizing freshwater ecosystems and their services. Watershed-level anthropogenic stress has negative effects on macroinvertebrate communities in lakes, streams and wetlands [1],[2],[3]. Development in the watershed is characterized by reduction/removal of natural vegetation, increased road density and increased proportion of other impervious surfaces, as well as higher human population density. The effects of development-associated stressors and those associated with agricultural land use on macroinvertebrate communities are mediated through increased nutrient loading, point and non-point source pollution, sediment loads, altered hydrologic and temperature regimes, and habitat destruction and fragmentation in riparian zones and littoral areas [1].

Several functional characteristics (*e.g.*, flow, drag or silt adaptations, respiration and locomotion techniques, feeding habits, voltinism, *reviewed in* [3]) are considered to be important indicators of the state of an ecosystem and its potential resilience to further anthropogenic modification. In particular, functional diversity (FD) is a critical property of a group of organisms at any scale because increased trait space breadth is likely to be associated with a greater diversity of ecosystem processes and nutrient pathways, which in turn increase resistance and resilience to perturbations [4],[5]. FD can be related to reticulation of the food web, which has important implications for the resilience of food webs as demonstrated in a theoretical study [6]. In terrestrial plant assemblages, greater functional diversity has been shown to maximize resource use in heterogeneous environments, and affect energy flow and ecosystem function (*reviewed in* [4], [7], [8]), and similar patterns were observed in theoretical studies [9]. However, little is known about the effects of reduced FD in littoral systems. Another important functional trait, voltinism, may have important implications for temporal redistribution of nutrient processing and ecosystem stability, and proportion of univoltine and other longer-lived organisms was shown to decrease with increasing land use stress [10]. Furthermore, changes in the relative abundance of different invertebrate functional groups can alter nutrient processing characteristics [11], [12], potentially triggering cascading effects in higher trophic levels, which may also affect littoral-pelagic and aquatic-terrestrial habitat coupling, by virtue of this group’s central position in aquatic food webs.

Although invertebrate functional trait responses to increasing anthropogenic stress have been described in streams-particularly changes in voltinism and respiration type [3] - those findings may not apply to lake and wetland systems because the effects of watershed-scale stressors are no longer mediated primarily through hydrological alteration and canopy clearing [1]. In addition, some of the more sensitive functional attributes, such as the proportion of semi- and merovoltine taxa and anoxia-intolerant taxa, are already under-represented in lentic systems because these systems routinely experience greater variability in parameters such as dissolved oxygen, temperature, and other factors [13]–[15]. It is important to understand whether functional attributes of littoral macroinvertebrate communities change in response to watershed-scale anthropogenic stress in order to design management and conservation strategies specific to freshwater littoral systems.

To assess the functional responses of lentic macroinvertebrates, we used wetland macroinvertebrate community composition data from the Great Lakes Environmental Indicators project [16] collected across a full gradient of watershed development stress (basin-wide minimum to maximum) to 1) test whether FD and the relative abundance of longer-lived (uni-, semi- and merovoltine) organisms are affected by watershed-level development and agriculture, and 2) investigate the functional changes in invertebrate communities. The *a priori* prediction was that FD and proportion of longer-lived taxa would decline with increasing development and agriculture due to an overall decrease in taxonomic diversity and reduced habitat availability resulting from the combined effects of direct habitat degradation and pollution commonly associated with these anthropogenic stressors [1]. Due to the large number of taxa and their wide range of physiologic tolerances and habitat requirements, macroinvertebrates can serve as an indicator of the changes impacting other assemblages, as well as a warning signal of changes in littoral food webs.

## Materials and Methods

**Figure 1:**
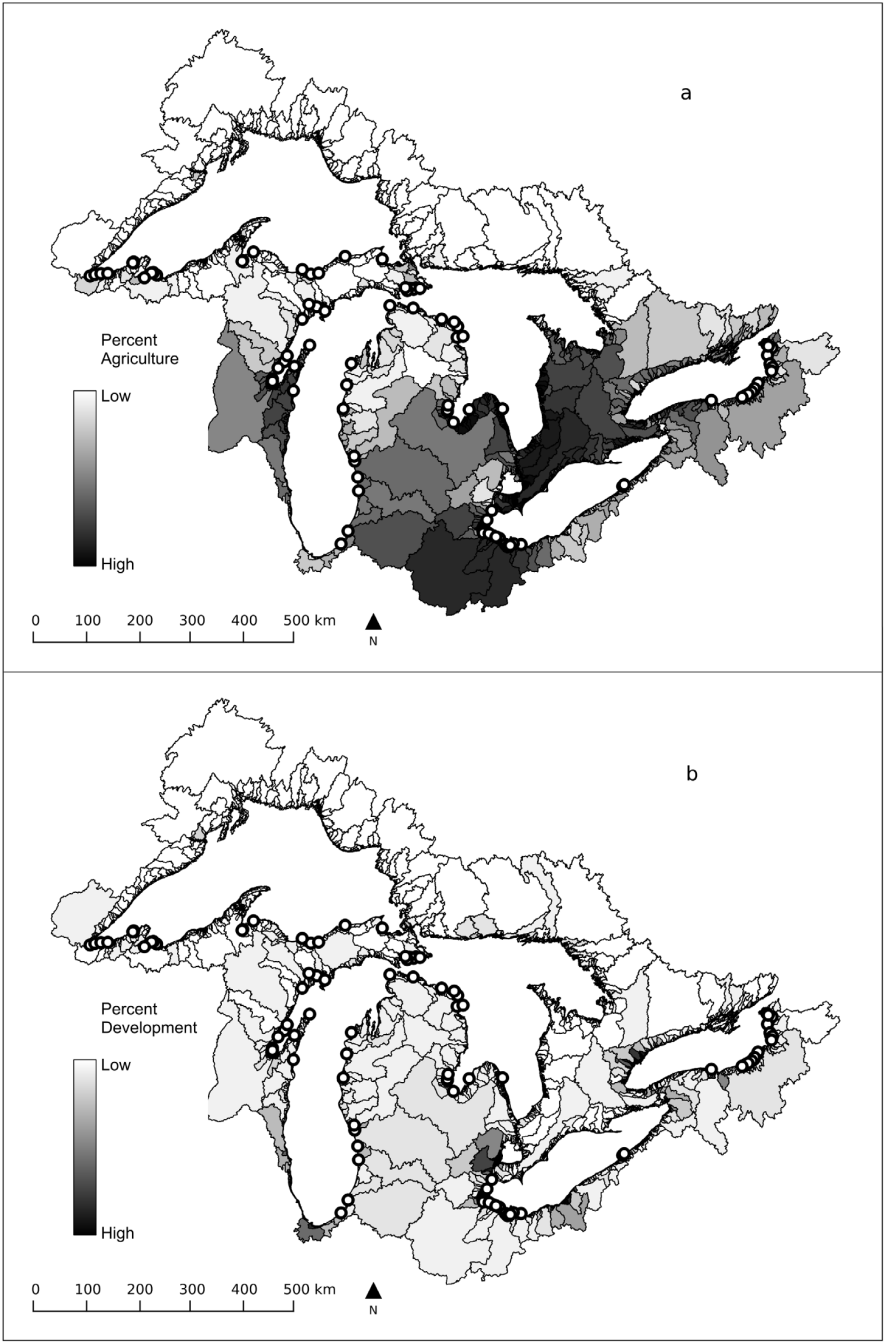
A map of the study sites in the coastal wetlands of the Laurentian Great Lakes, overlaid with the land-use stressor gradient for a) agriculture and b) development.

Macroinvertebrates were sampled in 101 coastal wetlands of the U.S. coastline of the Laurentian Great Lakes in the summer of 2002 and 2003 (Fig. 1). This dataset was previously collected for a different purpose, as part of a multidisciplinary effort to identify indicators of anthropogenic stress in the Great Lakes coastal zone (the Great Lakes Environmental Indicators project). No permits were required for macroinvertebrate sampling by the U.S. at the time of the original study and no recognized endangered or threatened invertebrate taxa occur in those coastal wetlands. All appropriate protected area sampling permits were secured by the original study.

The Laurentian Great Lakes include five glacial till lakes: Superior, Michigan, Huron, Erie and Ontario, located in the temperate part of North America. Lakes range from oligotrophic to eutrophic, with the surrounding land use spanning a gradient from completely unimpacted to highly impacted by anthropogenic activities including development and agriculture. Wetlands were equally distributed among lakes and across a full gradient of anthropogenic stress, previously defined as a composite of five major classes of anthropogenic pressure: agriculture, atmospheric deposition, land cover, human development, and point source pollution. The site selection procedure ensured representation of four geomorphic wetland types, including riverine, barrier-beach protected, and lacustrine coastal wetlands and embayments [17]. All wetlands were hydrologically connected to a Great Lake, and most wetlands had well-developed submerged and emergent macrophyte communities.

Proportion of human development and agriculture (by area) in a wetland’s watershed was derived from the USGS National Land Cover Dataset (2001) for each catchment delineated using ArcHydro with 10-m Digital Elevation Models (see [18] for details). Development was defined to include all residential, commercial and industrial areas, but did not include road density.

### Invertebrate sampling and functional metrics

To ensure the most complete sampling of the different habitats present within a wetland, macroinvertebrates were collected from all representative near shore land-use and shoreline (*e.g.*, sand beach, cobble beach, lawn, etc.) zones in each wetland. In each land-use-shoreline zone, two transects were extended perpendicular to shore, and macroinvertebrates were collected using 30-second D-frame dipnet sweeps at 0.25 and 0.75-m water depths along each transect. Sweeps were done through the water column from the bottom to the surface, in a forward direction parallel to the shore, regardless of vegetation type or presence. Samples were rinsed in a 250 µm sieve net or bucket to remove fine particles, and preserved in Kahle’s solution for laboratory processing. In the laboratory, macroinvertebrates were identified to the highest possible resolution (genus for most insects) using the most current keys available at that time [19], [20]. Data from the two transects in each zone were first averaged by depth, then averaged across zones to achieve site-level data. After taxonomic identification and sample averaging were completed, traits were assigned to each taxon using the latest reviews [14], [19], [21]–[27] and expert judgment (see Table S1 for details).

FD was measured using Rao’s Quadratic entropy (Q), which accounts for the relative abundances of species and for the functional differences between species by measuring differences between two randomly selected individuals with replacement (*see* [28], [29] for formulas and performance evaluation). It is closely correlated with the index of functional dispersion based on the centroid-distance approach [30], although it has also been demonstrated that this metric can be conservative under some scenarios due to negative covariation with species richness [29]. This analysis was performed with trait variables including trophic status, feeding mechanism, locomotion and primary and secondary functional feeding groups (Table S2), because those were the traits for which information was most consistently available across all encountered taxa. All traits were combined into a single Q-space.

The voltinism measure was expressed as the proportion of a sample comprised of taxa with long-lived aquatic phases (i.e. the proportion of individuals in a sample that were uni-, semi or merovoltine) to all other taxa. Voltinism is defined only for insect taxa; this life-history information was available for 77 taxa and unavailable for the remaining 85 insect taxa (of the total of 222 insect and non-insect taxa). This was mostly due to incomplete knowledge, as voltinism is one of the most difficult traits to describe, and to a lesser extent due to the level of resolution (variable life history at the species level, but identification to genus level) and differences in life history across a large geographic range of a taxon. Although all studies looking at voltinism unavoidably have this limitation, we chose to analyze this trait because it is a very important indicator of the state of macroinvertebrate assemblages and there is no *a priori* reason to suspect that the effect of this missing information is directional and not conservative on our ability to understand how longer-lived insects are affected by land use. Fifty-eight taxa in our samples were uni-, semi- or merovoltine, primarily belonging to Odonata and several genera of Trichoptera and Ephemeroptera, along with a few rarely occurring groups.

### Statistical Analyses

Multiple regression (MR) analyses were used to test for relationships between watershed stressor variables and voltinism and FD. Percent development was log-transformed, whereas percent agriculture was not transformed to satisfy the assumptions of regression residuals distribution, as tested using Shapiro-Wilk tests. To address the possibility of the confounding effect of sampling across large geographic scales, we conducted additional multiple regression analyses using latitude as a third predictor for each of the response variables. Latitude was significantly, but weakly correlated with agriculture (linear regression permutation p = 0.010, R^2^ = 0.06), but not with development (p = 0.26). Functional community structure was summarized using Principal Components Analysis (PCA) conducted on the log-transformed relative abundances of 12 functional traits. The resulting site Principal Component scores for each factor were regressed against the predictor variables as described above. Analyses were done in R 2.12.2 (R Development Core Team, Vienna, Austria), using packages *vegan* and *lattice*. Package *relaimpo* was used to calculate the “*lmg* relative importance metric” [31] for predictors in the multiple regression models, which produces averages of sequential sums of squares over all orderings of regressors (*see* [31] and package documentation for details). Although computationally intensive, the *lmg* procedure has been recommended because it decomposes R^2^ into non-negative contributions and accounts for direct effects as well as adjustments for other regressors in the model [32]. Natgrid, a two-dimensional random data interpolation package, was used to resample data to a regular grid and produce contour plots. Natgrid, based on nngridr package [33], implements a natural neighbour interpolation method and uses a weighted average method. The package was implemented through *matplotlib* [34], plotting library for the Python programming language.

## Results

**Figure 2:**
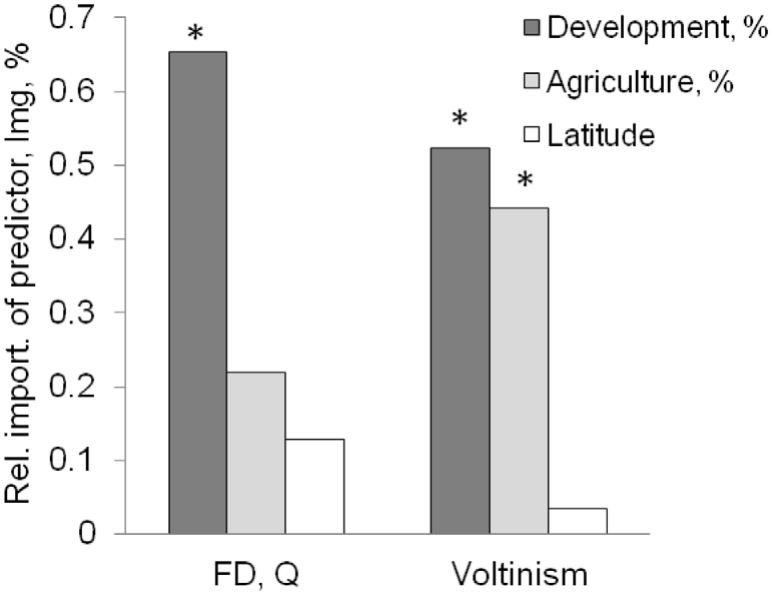
Relative importance of the two watershed stressors and latitude in explaining variation in the functional attributes of macroinvertebrate communities, as proportional *lmg* contribution. Note the overriding importance of development over agricultural stress and the lack of significant geographic confounding. Asterisks indicate predictors that were significant in the multiple regression (p<0.05).

**Figure 3:**
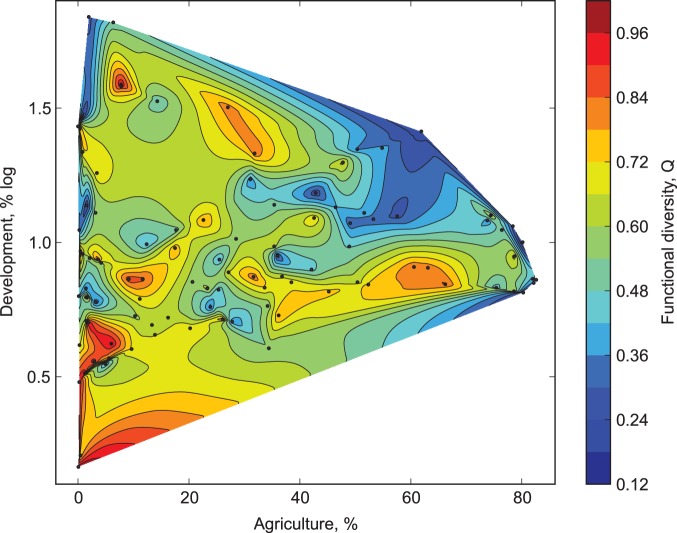
Macroinvertebrate functional diversity (Rao’s Q) as a function of development and agricultural stress in the watershed. Note that maximum values of the functional trait are observed mostly below a certain proportion of development (around mid-point of the y-axis, corresponding to 10% untransformed % development), indicating the over-riding contribution of that stressor; and if high values are observed at the higher levels of development, those occur only at sites with minimal agriculture. *[figure footnote] Contour lines divide the figure into a region where values are higher than they are on the contour line itself and a region where they are lower; values change across the line but not along the contour line, and the gradient is larger where contour lines are packed closer together.

Watershed development exerted stronger effects on all functional response variables than did watershed agriculture (Fig. 2). Rao’s functional diversity was significantly reduced by both development and agriculture (Fig. 3, MR F_2, 97_ = 9.60, adjusted R^2^ = 0.16, p = 0.003 and 0.004 for development and agriculture, respectively; see Table S3 for details and all regression coefficients). Development was the more important driver of this relationship (Fig. 2, Fig. S1a). To illustrate the extent of this effect, sites with more than 10% development had a 25% reduction in functional diversity compared to the less developed sites. The latitudinal predictor did not contribute significantly to the MR (p = 0.58), and its presence had almost no effect on the relative importance of the two stressors, confirming the lack of confounding effects (Table S3).

**Figure 4:**
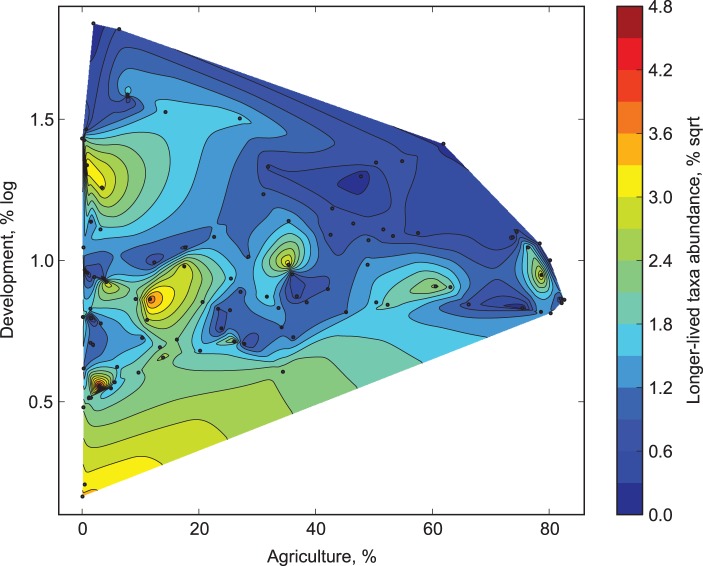
Relative abundance of long-lived taxa (uni-, semi- and merovoltine) as a function of development and agricultural stress in the watershed.

Relative abundance of those longer-lived taxa declined with increasing development in the watershed (MR F_2, 97_ = 11.17, adjusted R^2^ = 0.17; Fig. 4, p<0.001 for development, also Fig. S1b). The weak (Fig. 2) but significant negative effect of agriculture observed in the two-factor model (p = 0.017) was no longer significant (p = 0.062) when latitude was included as a predictor (see Table S3 for model details and regression coefficients).

The overall functional community structure was weakly but significantly affected by the extent of development in the watershed. The first PC axis, explaining 32% of the variation in functional community structure (eigenvalue = 3.74), was driven primarily by burrowers and filterers on one end vs. scrapers and clingers on the other end (*see* Table S4 for loadings). This axis was significantly positively correlated with percent development (MR F_2, 97_ = 5.82, adjusted R^2^ = 0.11; p = 0.004) but not with agriculture. In other words, the proportions of positively-loading groups, including burrowers and filterers, were positively correlated with greater amounts of watershed development, whereas scrapers and clingers were negatively correlated. This axis score tended to be negatively correlated with agriculture, although not significantly (p = 0.053), with this trend disappearing when we added latitude as a predictor, similar to the pattern observed for other response variables. The second PCA axis (eigenvalue = 3.10, percent variation explained = 19%) was mostly driven by omnivores (Table S4). This axis was weakly negatively correlated with percent agriculture (p = 0.011, R^2^ = 0.06).

## Discussion

This study demonstrates that macroinvertebrate functional diversity and abundance of longer-lived taxa in Laurentian Great Lakes coastal wetlands decreased significantly with greater watershed-level stress. Although macroinvertebrate communities are influenced by factors acting at many spatial scales, as has been demonstrated in a number of stream studies (e.g. [3], [35], [36] but see [37] for lake margins), our results indicate that the negative effects of watershed-level development were sufficiently robust to be detected against the background of a strong geographic gradient, the presence of additional stressors and a wide range of local habitat features. Compin and Céréghino [38] used self-organizing maps to show that, in human-modified landscapes, effects of stream watershed-scale stressors over-rode the influence of natural physical factors. It also means that large-scale stressors can produce detectable changes in ecosystem function, at least with respect to macroinvertebrate functional traits. The effect of watershed development was significant, but not highly predictive, reflecting the hierarchical complexity of the underlying factors affecting macroinvertebrate assemblages and leading to the high variability seen in many such datasets [15], [35].

A reduction in macroinvertebrate FD examined in this study translates into reduced community-level variation in foraging mechanisms and locomotion/substrate relations, which is likely to lead to significant alterations in food web structure and energy flow in those coastal wetland systems. Reduced FD is also likely to translate into decreased asynchrony of taxa responses to environmental perturbations (because more functionally similar taxa would have more synchronized responses to changes in resource abundance), which in turn has been proposed to be one of the key mechanisms reducing ecosystem stability in a theoretical modeling study [39].

The greater relative importance of development vs. agricultural stress is not surprising considering previously reported macroinvertebrate community thresholds at very low levels of development [40]–[44]. Similarly, a previous study found that development in the watershed was the best predictor of several macroinvertebrate metrics, including *Aeshna* abundance [37]. However, further study is needed to identify the more proximal factors driving this relationship, specifically in littoral systems. For instance, decline in longer-lived organisms could be mediated through the greater cumulative effects of development-associated stressors such as point-source pollution, in particular organic pollution, on these organisms, or through the destruction of biogenic (macrophyte) complexity, which has been shown to support greater abundance of several types of predators [45] and likely sustains more stable, and thereby more diverse, predator-prey populations [46]. It has proven difficult to elucidate repeatable patterns in these coastal wetlands, possibly due to their highly variable hydrology [35], [47], e.g. half a meter or more changes in water level on diel and annual basis due to fetch and climate change. Despite this, there is considerable interest in finding community metrics responsive to anthropogenic stress, and several such metrics have been found including Ephemeroptera-Trichoptera-Odonata richness-based metrics [48].

The observation of shift in functional community composition towards greater relative abundance of burrowers and filterers is consistent with other studies and has been previously related to increased sedimentation in streams [49]. Yet flashier hydrology and increased siltation, which are cited as the most common causes of anthropogenic changes in stream assemblages [1], are less likely to be the same factors responsible for observed changes in littoral assemblages, and lake-specific environmental variables responsible for these effects need to be investigated. Several functional groups (clingers, burrowers and insect filter-gatherers) were previously demonstrated to be affected by the extent of anthropogenic development in this system; however, responses were complex and dependent on several predictor variables as well as land use predictors’ buffer sizes [37].

Trait responses are generally less frequently reported in aquatic studies than trends in diversity and abundance [50]. Effects of watershed land use on FD have garnered even less attention, particularly in non-stream ecosystems. For lotic macroinvertebrates, it has been shown that trait type changed [51] and trait diversity decreased [52] with increasing agricultural land use. The latter study demonstrated that this effect was detectable both at the watershed scale and local patch scale, and was related to increasing sedimentation [52]. Significant effects on these important functional variables (*e.g.*, trait diversity) emphasize the need to further investigate functional responses to anthropogenic stress in lentic macroinvertebrates, the mechanisms potentially underlying these responses and the local factors that may mitigate those effects. Considering additional local factors as well as integrating traits with explicit consideration of trait linkages [53] may increase the predictive capability of future littoral trait models. However, what is of greater interest for future studies is that this uncertainty potentially reflects trait responses to smaller-scale habitat factors, which, if amendable to manipulation or restoration, could be used to ameliorate effects of watershed-scale land use.

In summary, we observed statistically and biologically significant reduction in macroinvertebrate FD and abundance of longer-lived taxa, and noticeable differences in functional community structure associated with increasing proportion of development in the watershed. These findings, along with the previously-observed threshold changes in macroinvertebrate community composition [44], show that lentic fauna exhibit significant functional changes associated with greater levels of watershed-scale anthropogenic stress.

## Supporting Information

Figure S1 **Univariate relationships with the development stressor.**
(DOCX)

Table S1 **List of observed taxa with taxonomic information and trait values.**
(XLSX)

Table S2 **List of possible states for each trait category used in calculating Rao’s Q.**
(DOCX)

Table S3 **Details of multiple regression analyses.**
(DOCX)

Table S4 **Trait loadings for the Principal Component Analysis.**
(DOCX)

## References

[1] Allan JD (2004) Landscapes and riverscapes: the influence of land use on stream ecosystems. Annu Rev Ecol Evol Syst 35: 257–284.

[2] Heino J (2010) Are indicator groups and cross-taxon congruence useful for predicting biodiversity in aquatic ecosystems? Ecol Indic 10: 112–117.

[3] Statzner B, Bêche L (2010) Can biological invertebrate traits resolve effects of multiple stressors on running water ecosystems? Freshw Biol 55: 80–119.

[4] Díaz S, Cabido M (2001) Vive la différence: plant functional diversity matters to ecosystem processes. Trends Ecol Evol 16: 644–655.

[5] Folke C, Carpenter SR, Walker BH, Scheffer M, Elmqvist T, et al (2004) Regime shifts, resilience and biodiversity in ecosystem management. Annu Rev Ecol Evol Syst 35: 557–581.

[6] Dunne JA, Brose U, Williams RJ, Martinez ND (2005) Modeling food web dynamics: complexity-stability implications. In: Belgrano A, Scharler UM, Dunne J, Ulanowics RE, editors. Aquatic food webs: an ecosystem approach. Oxford University Press, New York. 117–129.

[7] Hooper DU, Solan M, Symstad A, Diaz S, Gessner MO, et al.. (2002) Species diversity, functional diversity and ecosystem functioning. Chapter 17 in Loreau M, Naeem S, Inchausti P, editors. Biodiversity and Ecosystem Functioning: a Current Synthesis. Oxford University Press. 147–154.

[8] McGill BJ, Enquist BJ, Weiher E, Westoby M (2006) Rebuilding community ecology from functional traits. Trends Ecol Evol 21: 178–185. 16701083 10.1016/j.tree.2006.02.002

[9] Loreau M (1998) Biodiversity and ecosystem functioning: A mechanistic model. Proc Natl Acad Sci USA 95: 5632–5636. 9576935 10.1073/pnas.95.10.5632 PMC20430

[10] Dolédec S, Phillips N, Scarsbrook M, Riley RH, Townsend CR (2006) Comparison of structural and functional approaches to determining landuse effects on grassland stream invertebrate communities. J North Am Benthol Soc 25: 44–60.

[11] Covich AP, Palmer MA, Crowl TA (1999) The role of benthic invertebrate species in freshwater ecosystems. BioScience 49: 119–127.

[12] Cardinale BJ, Palmer MA, Collins SL (2002) Species diversity increases ecosystem functioning through interspecific facilitation. Nature 415: 426–429. 11807553 10.1038/415426a

[13] Merritt RW, Cummins KW, Berg MB, editors (2008) An Introduction to the Aquatic Insects of North America: Fourth Edition. Kendall/Hunt Publishing Company. 1214 p.

[14] Huryn AD, Wallace JB, Anderson NH (2008) Habitat, life history, secondary production and behavioral adaptations of aquatic insects. Chapter 5 in: Merritt RW, Cummins KW, Berg MB. An Introduction to the Aquatic Insects of North America. Kendall/Hunt Publishing Company. 55–104.

[15] Batzer DP (2013) The seemingly intractable ecological responses of invertebrates in North American wetlands: a review. Wetlands 33: 1–15.

[16] Niemi GJ, Kelly JR, Danz NP (2007) Environmental indicators for the coastal region of the North American Great Lakes: Introduction and prospectus. J Great Lakes Res 33: 1–12.

[17] Danz NP, Regal RR, Niemi GJ, Brady VJ, Hollenhorst TP, et al (2005) Environmentally stratified sampling design for the development of Great Lakes environmental indicators. Environ Monit Assess 102: 41–65. 15869177 10.1007/s10661-005-1594-8

[18] Hollenhorst TP, Johnson LB, Ciborowski J (2011) Monitoring land cover change in the Lake Superior basin. Aquat Ecosyst Health Manag 14: 433–442.

[19] Merritt RW, Cummins KW, editors (1995) An introduction to the aquatic insects of North America. Third edition. Kendall/Hunt Publishing Company. 862 p.

[20] Thorp JH, Covich AP, editors (2001) Ecology and classification of North American freshwater invertebrates. Second edition. Academic Press. 1056 p.

[21] Courtney GW, Merritt RW (2008) Aquatic Diptera Part One. Larvae of aquatic Diptera. Chapter 22 in: Merritt RW, Cummins KW, Berg MB. An Introduction to the Aquatic Insects of North America. Kendall/Hunt Publishing Company. 687–722.

[22] Morse JC, Holzenthal RW (2008) Trichoptera Genera. Chapter 18 in: Merritt RW, Cummins KW, Berg MB. An Introduction to the Aquatic Insects of North America. Kendall/Hunt Publishing Company. 481–552.

[23] Polhemus JT (2008) Aquatic and semiaquatic Hemiptera. Chapter 15 in: Merritt RW, Cummins KW, Berg MB. An Introduction to the Aquatic Insects of North America. Kendall/Hunt Publishing Company. 385–423.

[24] Solis MA (2008) Aquatic and semiaquatic Lepidoptera. Chapter 19 in: Merritt RW, Cummins KW, Berg MB. An Introduction to the Aquatic Insects of North America. Kendall/Hunt Publishing Company. 553–569.

[25] Tennessen KJ (2008) Odonata. Chapter 12 in: Merritt RW, Cummins KW, Berg MB. An Introduction to the Aquatic Insects of North America. Kendall/Hunt Publishing Company. 237–294.

[26] Waltz RD, Burian SK (2008) Ephemeroptera. Chapter 11 in: Merritt RW, Cummins KW, Berg MB. An Introduction to the Aquatic Insects of North America. Kendall/Hunt Publishing Company. 181–236.

[27] White DS, Roughly RE (2008) Aquatic Coleoptera. Chapter 20 in: Merritt RW, Cummins KW, Berg MB. An Introduction to the Aquatic Insects of North America. Kendall/Hunt Publishing Company. 571–671.

[28] Rao CR (1982) Diversity and dissimilarity coefficients – a unified approach. Theor Popul Biol 21: 24–43.

[29] Botta-Dukát Z (2005) Rao’s quadratic entropy as a measure of functional diversity based on multiple traits. J Veg Sci 16: 533–540.

[30] Laliberté E, Legendre P (2010) A distance-based framework for measuring functional diversity from multiple traits. Ecology 91: 299–305. 20380219 10.1890/08-2244.1

[31] Lindeman RH, Merenda PF, Gold RZ (1980) Introduction to Bivariate and Multivariate Analysis, Glenview, IL: Scott, Foresman. 444 p.

[32] Grömping U (2006) Relative importance for linear regression in R: the package relaimpo. J Stat Softw 17: 1–27.

[33] Watson D (1994) Nngridr-An Implementation of Natural Neighbor Interpolation. D. Watson. 170 p.

[34] Hunter JD (2007) Matplotlib: A 2D graphics environment. Comput Sci Eng 9: 90–95.

[35] Poff NL (1997) Landscape filters and species traits: towards mechanistic understanding and prediction in stream ecology. J North Am Benthol Soc 16: 391–409.

[36] Weigel BM, Wang L, Rasmussen PW, Butcher JT, Stewart PM, et al (2003) Relative influence of variables at multiple spatial scales on stream macroinvertebrates in the Northern Lakes and Forest ecoregion, U.S.A. Freshw Biol. 48: 1440–1461.

[37] Brazner JC, Danz NP, Niemi GJ, Regal RR, Hollenhorst T, et al (2007) Responsiveness of Great Lakes wetland indicators to human disturbances at multiple spatial scales: a multi-assemblage assessment. J Great Lakes Res 33: 42–66.

[38] Compin A, Céréghino R (2007) Spatial patterns of macroinvertebrate functional feeding groups in streams in relation to physical variables and land-cover in Southwestern France. Landsc Ecol 22: 1215–1225.

[39] Loreau M, de Mazancourt C (2013) Biodiversity and ecosystem stability: a synthesis of underlying mechanisms. Ecol Lett 16: 106–115. 23346947 10.1111/ele.12073

[40] King RS, Baker ME (2010) Considerations for analyzing ecological community thresholds in response to anthropogenic environmental gradients. J North Am Benthol Soc 29: 998–1008.

[41] Hilderbrand RH, Utz RM, Stranko SA, Raesly RL (2010) Applying thresholds to forecast potential biodiversity loss from human development. J North Am Benthol Soc 29: 1009–1016.

[42] Utz RM, Hilderbrand RH (2011) Interregional variation in urbanization-induced geomorphic change and macroinvertebrate habitat colonization in headwater streams. J North Am Benthol Soc 30: 25–37.

[43] Kail J, Arle J, Jähnig SC (2012) Limiting factors and thresholds for macroinvertebrate assemblages in European rivers: Empirical evidence from three datasets on water quality, catchment urbanization, and river restoration. Ecol Indic 18: 63–72.

[44] Kovalenko KE, Brady VJ, Brown TN, Ciborowski JJH, Danz NP, et al. (2014) Congruence of community thresholds in response to anthropogenic stressors in Great Lakes coastal wetlands. Freshw Sci (In press).

[45] Heino J (2008) Patterns of functional biodiversity and function–environment relationships in lake littoral macroinvertebrates. Limnol Oceanogr 53: 1446–1455.

[46] Kovalenko KE, Thomaz SM, Warfe DM (2012) Habitat complexity: approaches and future directions. Editorial review. Hydrobiologia 685: 1–17.

[47] Gathman JP, Burton TM (2011) A Great Lakes coastal wetland invertebrate community gradient: relative influence of flooding regime and vegetation zonation. Wetlands 31: 329–341.

[48] Uzarski DG, Burton TM, Genet JA (2004) Validation and performance of an invertebrate index of biotic integrity for Lakes Huron and Michigan fringing wetlands during a period of lake level decline. Aquat Ecosyst Health Manag 7: 269–288.

[49] Larsen S, Pace G, Ormerod SJ (2011) Experimental effects of sediment deposition on the structure and function of macroinvertebrate assemblages in temperate streams. River Res Appl 27: 257–267.

[50] Stendera S, Adrian R, Bonada N, Cañedo-Argüelles M, Hugueny B, et al (2012) Drivers and stressors of freshwater biodiversity patterns across different ecosystems and scales: a review. Hydrobiologia 696: 1–28.

[51] Richards C, Haro RJ, Johnson LB, Host GE (1997) Catchment and reach-scale properties as indicators of macroinvertebrate species traits. Freshw Biol 37: 219–230.

[52] Larsen S, Ormerod SJ (2010) Combined effects of habitat modification on trait composition and species nestedness in river invertebrates. Biol Conserv 143: 2638–2646.

[53] Verberk WCEP, van Noordwijk CGE, Hildrew AG (2013) Delivering on a promise: integrating species traits to transform descriptive community ecology into a predictive science. Freshw Sci 32: 531–547.

