# Supplementary material for: Functional Changes in Littoral Macroinvertebrate Communities in Response to Watershed-Level Anthropogenic Stress
Source: PLoS One. 2014 Jul 9;9(7):e101499. doi: 10.1371/journal.pone.0101499 (PMC4090147; doi:10.1371/journal.pone.0101499)

**Figure S1. Univariate relationships with the development stressor**

**S**imple linear relationship with the most important predictor, % Development, for A) macroinvertebrate functional diversity (p = 0.002, r = -0.30) and B) relative abundance of long-lived (uni-, semi- and merovoltine) taxa (p < 0.001, r = -0.37).


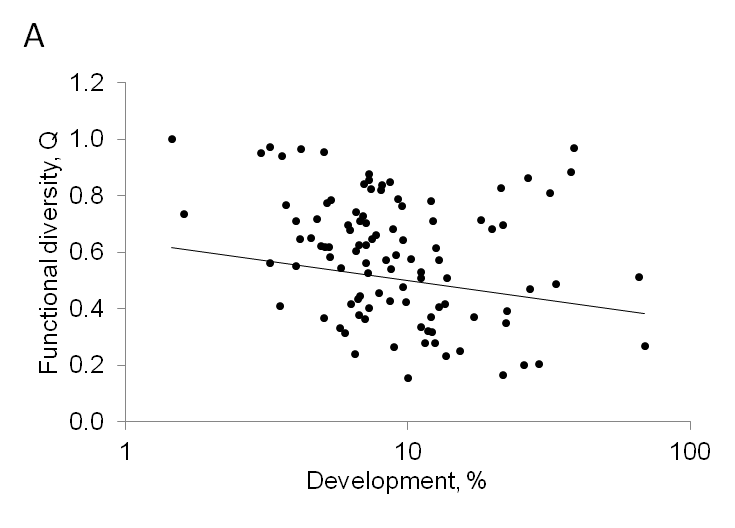

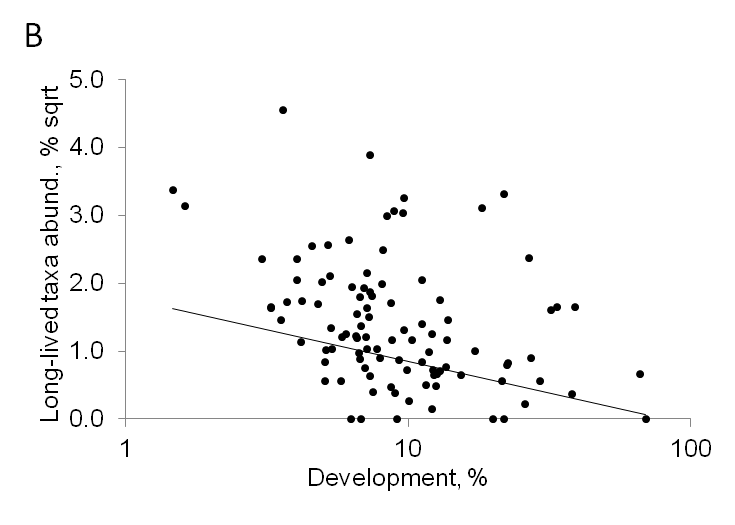

Supplement: Figure S1 — Univariate relationships with the development stressor. (DOCX) [file pone.0101499.s001.docx]
