# Supplementary material for: Functional Changes in Littoral Macroinvertebrate Communities in Response to Watershed-Level Anthropogenic Stress
Source: PLoS One. 2014 Jul 9;9(7):e101499. doi: 10.1371/journal.pone.0101499 (PMC4090147; doi:10.1371/journal.pone.0101499)
Supplement: Table S2 — List of possible states for each trait category used in calculating Rao’s Q. (DOCX) [file pone.0101499.s003.docx]

**Table S2. List of possible states for each trait category used in calculating Rao’s Q**

The range of functional traits assessed in this study was narrower than those considered in most stream studies, partly due to the limited availability of trait values for many North American macroinvertebrates (Statzner and Bêche 2010), and partly because some traits are likely to have limited application to stress assessment in lentic environments (e.g., flow and drag adaptations).

| **Trait category** | **Possible states** |
| --- | --- |
|  |  |
| Trophic Status | carnivore, detritivore, herbivore, omnivore |
| Functional Group (primary) | collector, grazer, parasite, piercer, predator, shredder |
| Functional Group (secondary) | collector, grazer, parasite, piercer, predator, shredder |
| Feeding mechanism (primary) | chewer, carnivore-piercer, engulfer, filterer, gatherer, herbivore-piercer, internal parasite, scraper |
| Food Source | coarse POM, fine POM, living animal tissue, living vascular plants, periphyton |
| Locomotion/substrate relations | burrower, climber, clinger, planktonic, skater, attached, sprawler, swimmer |
|  |  |

* We selected traits which both sufficiently describe the occupied trait space and for which the most complete information is available based on the most recent sources (Merritt et al. 2008).

Majority of missing values are in the secondary functional group category, and those are true null values (i.e. no secondary functional group exists) as opposed to missing information. In the minority of cases, the values are truly missing due to either lack of information or presence of species within a genus with different trait values.

Functional diversity metrics are well-equipped to cope with the problem of missing values. As discussed in Laliberte and Legendre (2010), the index developed by Villéger et al. (2008) is sensitive to missing values, whereas FD metric proposed by Laliberte and Legendre 2010 and the closely related original Rao’s Q tolerate missing values.
