# Supplementary material for: Functional Changes in Littoral Macroinvertebrate Communities in Response to Watershed-Level Anthropogenic Stress
Source: PLoS One. 2014 Jul 9;9(7):e101499. doi: 10.1371/journal.pone.0101499 (PMC4090147; doi:10.1371/journal.pone.0101499)
Supplement: Table S3 — Details of multiple regression analyses. (DOCX) [file pone.0101499.s004.docx]

**Table S3. Details of multiple regression analyses**

**a) functional diversity (Rao’s Q)**

**1) with latitude**

lm(formula = Q ~ log(%Dev) + %Ag + Lat)

**Coefficients**

Estimate Std. Error t value Pr(>|t|)

(Intercept) 6.855e-01 2.878e-01 2.382 0.01918 *

log(%Dev) -8.938e-02 2.913e-02 -3.069 0.00279 **

%Ag -2.106e-03 7.959e-04 -2.646 0.00951 **

Lat 6.344e-08 1.159e-07 0.547 0.58547

Signif. codes: 0 ‘***’ 0.001 ‘**’ 0.01 ‘*’ 0.05 ‘.’ 0.1 ‘ ’ 1

Residual standard error: 0.1997 on 96 degrees of freedom, Multiple R-squared: 0.1678, Adjusted R-squared: 0.1417, F-statistic: 6.45 on 3 and 96 DF, p-value: 0.0005028

**Relative importance metrics**

lmg last first pratt

log(%Dev) 0.52302241 0.56321948 0.49670743 0.52397057

%Ag 0.44222625 0.41886938 0.46338919 0.44870385

Lat 0.03475134 0.01791114 0.03990337 0.02732558

**2) without latitude**

lm(formula = Q ~ log(%Dev) + %Ag)

**Coefficients**

Estimate Std. Error t value Pr(>|t|)

(Intercept) 0.8385261 0.0680789 12.317 < 2e-16 ***

log(%Dev) -0.0871347 0.0287307 -3.033 0.00311 **

%Ag -0.0022238 0.0007637 -2.912 0.00446 **

Signif. codes: 0 ‘***’ 0.001 ‘**’ 0.01 ‘*’ 0.05 ‘.’ 0.1 ‘ ’ 1

Residual standard error: 0.1989 on 97 degrees of freedom, Multiple R-squared: 0.1652, Adjusted R-squared: 0.1479, F-statistic: 9.595 on 2 and 97 DF, p-value: 0.0001577

**Relative importance metrics**

calc.relimp(fit,type=c("lmg","last","first","pratt"), rela=TRUE)

lmg last first pratt

log(%Dev) 0.5187187 0.5203199 0.5173515 0.5188359

%Ag 0.4812813 0.4796801 0.4826485 0.4811641

**b) Voltinism**

**1) with latitude**

lm(formula = Volt ~ log(%Dev) + %Ag + Lat)

**Coefficients**

Estimate Std. Error t value Pr(>|t|)

(Intercept) 5.999e-01 1.221e+00 0.492 0.6242

log(%Dev) -5.067e-01 1.235e-01 -4.102 8.6e-05 ***

%Ag -6.371e-03 3.376e-03 -1.887 0.0622 .

Lat 8.577e-07 4.917e-07 1.744 0.0843 .

Signif. codes: 0 ‘***’ 0.001 ‘**’ 0.01 ‘*’ 0.05 ‘.’ 0.1

Residual standard error: 0.8468 on 96 degrees of freedom, Multiple R-squared: 0.2121, Adjusted R-squared: 0.1875, F-statistic: 8.617 on 3 and 96 DF, p-value: 4.024e-05

**Relative importance metrics**

lmg last first pratt

log(%Dev) 0.6531803 0.7181117 0.6031302 0.6592516

%Ag 0.2191636 0.1520154 0.2739794 0.2101756

Lat 0.1276561 0.1298728 0.1228903 0.1305729

**2) without latitude**

lm(formula = Volt~ log(%Dev) + %Ag)

**Coefficients**

Estimate Std. Error t value Pr(>|t|)

(Intercept) 2.668048 0.292832 9.111 1.11e-14 ***

log(%Dev) -0.476334 0.123581 -3.854 0.000208 ***

%Ag -0.007958 0.003285 -2.423 0.017263 *

Signif. codes: 0 ‘***’ 0.001 ‘**’ 0.01 ‘*’ 0.05 ‘.’ 0.1

Residual standard error: 0.8557 on 97 degrees of freedom, Multiple R-squared: 0.1872, Adjusted R-squared: 0.1704, F-statistic: 11.17 on 2 and 97 DF, p-value: 4.313e-05

**Relative importance metrics**

lmg last first pratt

log(%Dev) 0.7011706 0.7168124 0.6876338 0.7024292

%Ag 0.2988294 0.2831876 0.3123662 0.2975708
