# Supplementary material for: Functional Changes in Littoral Macroinvertebrate Communities in Response to Watershed-Level Anthropogenic Stress
Source: PLoS One. 2014 Jul 9;9(7):e101499. doi: 10.1371/journal.pone.0101499 (PMC4090147; doi:10.1371/journal.pone.0101499)
Supplement: Table S4 — Trait loadings for the Principal Component Analysis. (DOCX) [file pone.0101499.s005.docx]

**Table S4. Trait loadings for the Principal Component Analysis**

PCA of 12 macroinvertebrate functional groups in 101 sites in Great Lakes coastal wetlands. The first three axes were significant (p = 0.001 for each, other axes were not significant p > 0.7; based on 999 randomizations; eigenvalues for the first three axes were 3.74, 3.10 and 2.28). Only the first two axes were discussed in the paper due to the small amount variation explained by the third axis (16%) and absence of strongly loading groups for this axis.

| **Group** | **Axis 1** | **Axis 2** | **Axis 3** |
| --- | --- | --- | --- |
|  |  |  |  |
| Burrower | **-0.875** | -0.207 | -0.098 |
| Filterer | **-0.872** | 0.170 | 0.294 |
| Detritiv | -0.486 | **-0.622** | 0.579 |
| InsectFi | -0.434 | 0.442 | -0.577 |
| Omnivore | -0.152 | **0.868** | -0.367 |
| Sprawler | -0.140 | 0.041 | 0.007 |
| Climber | 0.389 | -0.386 | -0.205 |
| Predator | 0.453 | -0.566 | -0.530 |
| Swimmer | 0.482 | -0.303 | -0.544 |
| Shredder | 0.483 | 0.072 | 0.448 |
| Clingers | **0.685** | 0.447 | 0.455 |
| Scraper | **0.702** | 0.294 | 0.287 |
|  |  |  |  |
